# Ophthalmological care delivery in East Kazakhstan: a qualitative study of ophthalmologists on system barriers and service organization

**DOI:** 10.3389/fpubh.2026.1822513

**Published:** 2026-05-20

**Authors:** Manas Tulekenov, Gulnara Kapanova, Aziza Imamatdinova, Karlygash Zhybanisheva, Zhanna Sarbassova, Kenesh Dzhusupov

**Affiliations:** 1 Farabi University, Almaty, Kazakhstan; 2 Asfendiyarov Kazakh National Medical University, Almaty, Kazakhstan; 3 Kazakh-Russian Medical University, Almaty, Kazakhstan; 4 International Higher School of Medicine, Bishkek, Kyrgyzstan; 5 Osh State University, Osh, Kyrgyzstan

**Keywords:** healthcare accessibility, Kazakhstan, ophthalmologic care, ophthalmologists, primary healthcare, qualitative research

## Abstract

**Background:**

Access to ophthalmologic care is essential for maintaining eye health and preventing vision loss. However, barriers within health systems may limit timely access to services. Understanding these barriers from the perspective of healthcare providers may help improve the organization and accessibility of ophthalmological care.

**Methods:**

A qualitative study was conducted in the East Kazakhstan region using semi-structured interviews with 22 ophthalmologists working in primary healthcare and hospital settings. Interviews explored public awareness, workforce capacity, service provision, equipment availability, and system-level barriers. Data were analyzed using thematic analysis supported by Dedoose software.

**Results:**

Five major themes were identified: limited public awareness of eye diseases, workforce shortages and uneven distribution of ophthalmologists, gaps in equipment and resource availability in primary healthcare, systemic barriers such as long waiting times and referral delays, and proposed strategies for improving ophthalmological services. Participants emphasized that shortages of specialists and diagnostic equipment at the primary healthcare level contribute to delayed diagnosis and increased reliance on hospital-based care.

**Conclusion:**

Improving ophthalmological care in East Kazakhstan requires strengthening primary healthcare capacity, increasing workforce availability, expanding preventive programs, and improving infrastructure and referral pathways. Addressing these challenges may improve early detection of eye diseases and enhance access to ophthalmic services.

## Introduction

The prevalence of eye diseases is increasing globally, primarily due to an aging population and lifestyle changes (1). Recent studies show a rise in glaucoma cases; in 2022, the global incidence rate of primary open-angle glaucoma was 23.46 per 10,000 person-years among individuals aged 40–79 (2). The global burden of eye diseases is increasing, driven by population aging and changing lifestyles. Conditions such as glaucoma, cataracts, and retinal disorders remain major contributors to visual impairment and blindness worldwide. Early detection through regular ophthalmologic care is essential for preventing avoidable vision loss (3, 4).

Despite the importance of early detection, access to ophthalmological care remains uneven across regions. Barriers to care include affordability, accessibility, limited awareness, and health system constraints. Previous studies have also identified challenges such as transportation difficulties, time constraints, and gaps between provider and patient perceptions of eye care needs (5–8). These barriers contribute to delayed care-seeking and reduced utilization of preventive services.

In Kazakhstan, the burden of eye diseases, including glaucoma and cataracts, is increasing (9). Ophthalmological services are formally integrated into the national healthcare system and are provided through the State Fund for Free Medical Care and Compulsory Medical Insurance, as well as through out-of-pocket services. National regulations define workforce standards and the scope of ophthalmological care across outpatient and inpatient settings, including prevention, diagnostics, treatment, and long-term monitoring (10).

Despite the presence of this regulatory framework, access to ophthalmological care remains uneven. Services are concentrated in large urban centers such as Almaty and Astana, where specialist availability and infrastructure are more developed. In contrast, regional and rural areas may face challenges related to workforce distribution, resource availability, and service organization, potentially limiting timely access to care.

The East Kazakhstan region, characterized by a combination of industrial and rural environments, provides a relevant setting for exploring these challenges. Environmental factors, including industrial pollution, as well as geographic disparities in healthcare access, may influence both the burden of eye diseases and access to ophthalmological services. At the same time, the organization of care across urban and rural settings may shape how services are delivered and utilized.

While previous research has primarily examined barriers to ophthalmic care from patient perspectives, less attention has been given to the experiences of frontline ophthalmologists working within regional health systems, particularly in post-Soviet and middle-income contexts. Understanding provider perspectives is important for identifying structural and organizational barriers that may not be fully captured in patient-based studies.

This study aims to explore ophthalmologists’ perspectives on the accessibility and organization of ophthalmological services in the East Kazakhstan region, with a focus on identifying perceived barriers to care. By examining provider perspectives within a geographically diverse setting, this study contributes to understanding health system constraints affecting access to specialized care in transitional healthcare systems.

## Methods

This study employed a qualitative descriptive design using semi-structured interviews and thematic analysis to explore ophthalmologists’ perspectives on the organization and delivery of ophthalmic care in the East Kazakhstan region. The study was conducted and reported in accordance with the Consolidated Criteria for Reporting Qualitative Research (COREQ) and the Standards for Reporting Qualitative Research (SRQR).

### Interview guide development

The semi-structured interview guide was developed by a multidisciplinary research team based on health systems and implementation frameworks. It included six predefined domains: patient awareness, human resources, service delivery, infrastructure, health information systems, and barriers/solutions. The guide was piloted with two ophthalmologists to ensure clarity and relevance, and minor refinements were made. The guide functioned as a flexible qualitative tool rather than a standardized instrument.

### Data collection

Interviews were conducted between December 2024 and March 2025 by the first author. Due to participants’ reluctance toward audio recording, interviews were documented using structured written records completed during the interview. Responses were recorded as close to verbatim as possible and were immediately reviewed and confirmed with participants (member checking) to enhance accuracy.

Field notes were expanded immediately after each interview to capture contextual details and non-verbal cues. These written records and field notes were subsequently consolidated into structured textual datasets for analysis. We acknowledge that this approach differs from conventional audio-recorded interviews and may limit the capture of fully verbatim language. As such, the data represents near-verbatim reconstructions rather than complete transcripts, and this has been considered in the analysis and interpretation.

Basic participant characteristics related to professional experience and workplace setting were collected. Workplace setting was categorized into urban PHC, urban hospital-based services, and rural hospital settings that also provide PHC. More detailed demographic variables (e.g., age, sex) were not systematically recorded as part of the study design.

### Sampling

Purposive sampling was used to recruit ophthalmologists from both primary care (PHC) and hospital settings. A total of 22 participants were included. Sampling was guided by the aim of capturing diverse perspectives across healthcare levels and domains relevant to ophthalmic care delivery. Recruitment continued until thematic redundancy was observed, with no substantially new themes emerging.

### Data preparation and analytic approach

Written interview records and expanded field notes were treated as the primary data source. These materials were reviewed, cleaned, and organized into analyzable text immediately following each interview. The unit of analysis was defined as a meaningful segment of text (sentence or phrase) expressing a single idea, perception, or experience related to ophthalmic care. These steps formed a structured analytic workflow linking data collection, preparation, and interpretation.

We acknowledge that some level of interpretive filtering occurred during note-taking, as responses were documented in real time. To mitigate this, responses were verified with participants during the interview, and analytic interpretations were continuously revisited during coding.

### Data analysis

Data were analyzed using thematic analysis supported by Dedoose software. A hybrid coding approach was applied, combining deductive codes based on predefined domains and inductive codes emerging from the data. Three researchers independently coded the dataset. Coding consistency was ensured through iterative comparison, and discrepancies were resolved through consensus discussions. The codebook was refined iteratively throughout the analytical process. To address the absence of audio transcripts, analytical rigor was strengthened through: Researcher triangulation; Audit trail documentation; Peer debriefing. Continuous comparison between raw notes and coded interpretations.

## Results

A total of 22 ophthalmologists participated in the study, representing diverse practice settings across the East Kazakhstan region. Participants were distributed across urban primary healthcare (*n* = 8), urban hospital-based services (*n* = 10), and rural hospital settings with integrated primary care functions (*n* = 4). Participants represented a range of practice contexts, including urban and rural settings as well as both primary and hospital-based care. While the analysis was not structured as a formal comparison between these groups, some variations in how challenges were described were noted across different service contexts.

Participants had between 1 and 25 years of professional experience in primary healthcare (mean: 12.1 years) and between 1 and 15 years in hospital settings (mean: 8.0 years). Participant characteristics are summarized in Table 1.

**Table 1:** Participant characteristics.

Characteristic	Category	Number (*n* = 22)
Workplace setting	Primary healthcare (PHC)	8
	Hospital	10
	Rural Hospital with PHC	4
Years of professional experience (PHC)	1–10 years	5
	11–20 years	4
	>20 years	3
Years of professional experience (Hospital)	1–5 years	4
	6–10 years	4
	>10 years	2
Mean years of experience (SD not available)	PHC	12.1
	Hospital	8.0

While all participants discussed similar systemic challenges, important differences emerged between PHC and hospital settings, as well as variation in how barriers were experienced and interpreted. Five major themes were identified: (1) public awareness and prevention, (2) workforce capacity and competencies, (3) equipment and resources, (4) systemic access barriers, and (5) improvement strategies. Although many perspectives were shared across participants, differences in emphasis and interpretation of barriers were also evident. Importantly, these themes were not independent; rather, participants described interconnected structural constraints, where workforce shortages, equipment limitations, and referral inefficiencies reinforced one another. Quotations presented below are near-verbatim reconstructions based on written records and are edited minimally for clarity while preserving meaning. The themes and corresponding subthemes are summarized in Table 2.

**Table 2:** Main themes and subthemes identified from thematic analysis.

Theme	Subthemes	Example quote
Public awareness and prevention	Limited awareness of eye diseases; insufficient educational programs; delayed care-seeking	“Many people simply do not know when they should visit an ophthalmologist.” (Physician 9)
Workforce capacity and competencies	Shortage of ophthalmologists in PHC; uneven distribution of specialists; high workload	“Some primary healthcare organizations simply do not have an ophthalmologist.” (Physician 11)
Equipment and resource availability	Limited diagnostic equipment in PHC; restricted medication availability	“Some PHC organizations lack the necessary ophthalmic equipment, which limits early detection.” (Physician 7)
System barriers to care	Long waiting times; overcrowded clinics; insufficient hospital capacity	“There are often long queues for ophthalmology appointments.” (Physician 2)
Proposed solutions	Screening programs; workforce expansion; equipment investment; public education	“If we had more specialists and better equipment, patients would not need to wait so long.” (Physician 8)

### Theme 1: limited public awareness and preventive eye care

Participants consistently described public awareness of eye diseases as moderate to low, indicating a gap between existing preventive programs and population engagement. While some acknowledge that patients increasingly access health information through the internet, most physicians believed that knowledge about eye disease prevention and the importance of regular ophthalmologic examinations remains limited.

Preventive initiatives such as screenings and routine eye examinations are implemented within the healthcare system, particularly targeting older adults and young children. However, respondents noted that these programs often fail to reach working-age adults, who may not seek care until symptoms become severe.

One physician emphasized the need for stronger educational efforts to improve public understanding of eye health:

“*There should be more educational programs for the population. Many people simply do not know when they should visit an ophthalmologist*.” (Physician 9).

Several also highlighted organizational challenges affecting the effectiveness of prevention programs, including limited resources and inconsistent implementation across healthcare facilities.

*“The organization of prevention programs is not always strong, which may be related to a lack of resources or insufficient coverage of certain population groups.”* (Physician 3).

Although outreach initiatives such as screenings and regional medical visits are conducted, not all respondents were fully aware of how these programs are coordinated at the system level. This lack of clarity suggests potential communication gaps within the healthcare system regarding preventive strategies.

Overall, these findings suggest that preventive strategies are present but insufficiently integrated into routine care and fail to effectively engage key population groups, particularly working-age individuals. However, not all participants viewed awareness levels in the same way, with some suggesting that access to online information has improved patient knowledge, while others emphasized persistent gaps in understanding and delayed care-seeking.

### Theme 2: workforce capacity and professional competencies

A major concern raised by respondents was the uneven distribution of ophthalmologists across healthcare settings. While hospital-based ophthalmology services were generally perceived as adequately staffed and professionally competent, many primary healthcare facilities face shortages of ophthalmologists.

Participants reported that this imbalance often leads patients to seek care directly at hospitals or specialized urban centers rather than local primary healthcare clinics. Physicians described how staff shortages and heavy workloads in PHC facilities limit the ability to provide timely consultations and follow-up care.

*“Some primary healthcare organizations simply do not have an ophthalmologist, which creates problems with access to specialized care.”* (Physician 11).

In addition to workforce shortages, several respondents noted that patients frequently attempt to manage eye problems independently before seeking professional medical care. Self-medication and reliance on pharmacy consultations were reported as common behaviors that may delay diagnosis.

*“Patients often try to treat eye problems themselves or ask pharmacists for advice before coming to us, which sometimes leads to complications.”* (Physician 6).

Opinions regarding the competencies of ophthalmologists in primary healthcare settings were mixed. While many participants considered the level of clinical competence to be adequate, others emphasized gaps in training and the need for further professional development. This reflects differing interpretations of workforce capacity, suggesting that the perceived quality of care may depend on local conditions and expectations, including differences in resource availability and service organization, particularly in smaller or resource-limited settings.

The competencies of ophthalmology nurses also varied across settings. In primary healthcare facilities, some nurses had transitioned from other medical specialties and may not have received specialized ophthalmology training.

*“Some nurses previously worked in other departments and later switched to ophthalmology, which may affect the quality of care.”* (Physician 5).

In contrast, respondents expressed strong confidence in the qualifications and professional experience of ophthalmologists and nurses working in hospital environments.

This imbalance reflects a structural misalignment between workforce distribution and patient demand, contributing to bypassing of PHC and overreliance on hospital services. These patterns suggest that workforce challenges may manifest differently depending on service context, with primary care settings experiencing more pronounced constraints related to staffing and workload.

### Theme 3: equipment and resource availability

Access to diagnostic equipment and medical technologies emerged as another important theme influencing the quality of ophthalmological care. While many respondents described the availability of equipment in hospital settings as satisfactory, challenges were more frequently reported at the primary healthcare level. Several physicians indicated that certain PHC facilities lack modern ophthalmic diagnostic tools necessary for early detection of eye diseases.

*“Some PHC organizations lack the necessary ophthalmic equipment, which limits our ability to detect diseases early.”* (Physician 7).

Participants also noted that although technical staff are usually responsible for maintaining equipment, some healthcare workers were uncertain about procedures for addressing equipment malfunctions.

*“If equipment breaks down, it can take time before it is repaired or replaced, especially in smaller facilities.”* (Physician 14).

Regarding medication availability, most reported that essential ophthalmic medications are generally accessible, particularly for patients receiving surgical treatment. However, the selection of medications was often limited to more cost-effective options. Some physicians also described inconsistencies in the distribution of ophthalmic medications within healthcare organizations.

*“Sometimes medications are available only if the patient purchases them independently, which can create difficulties for some patients.”* (Physician 10).

These challenges suggest that resource limitations may affect the consistency and efficiency of ophthalmological care, particularly in primary healthcare settings.

Together, these barriers reflect systemic inefficiencies in care coordination and resource allocation, limiting timely access to ophthalmological services. These findings indicate that resource limitations are more frequently experienced at the primary care level, potentially influencing the capacity for early detection and referral.

### Theme 4: system-level barriers to accessing ophthalmological care

Respondents identified several systemic barriers that limit patient access to ophthalmological services. The most frequently mentioned challenges included long waiting times, overcrowded outpatient clinics, and insufficient hospital bed capacity.

*“There are often long queues for ophthalmology appointments, especially in primary healthcare clinics.”* (Physician 2).

In many cases, patients are referred from primary healthcare facilities to specialized ophthalmology centers due to limited local capacity. In addition to structural barriers, respondents emphasized challenges related to health information management. Several physicians reported that the lack of comprehensive medical records for patients with ophthalmological conditions can hinder early detection and long-term monitoring of eye diseases.

*“Without proper medical records and monitoring systems, it becomes difficult to detect eye diseases early.”* (Physician 12).

Limited financial resources and the need for multiple diagnostic tests before treatment were also cited as factors contributing to delays in care.

Together, these barriers reflect systemic inefficiencies in care coordination and resource allocation, limiting timely access to ophthalmological services. Participants’ descriptions suggest that these barriers are interconnected and do not operate independently but instead form a reinforcing system, where limitations at the primary care level contribute to increased demand for hospital-based services, which in turn exacerbates waiting times and resource constraints.

### Theme 5: strategies for improving ophthalmological services

Participants proposed several strategies to improve the accessibility and quality of ophthalmological care in the region. The most frequently suggested interventions included expanding screening programs, increasing the number of ophthalmologists, and investing in modern diagnostic equipment.

*“If we had more specialists and better equipment, patients would not need to wait so long for consultations.”* (Physician 8).

Respondents also emphasized the importance of strengthening public education campaigns to increase awareness of eye disease prevention and encourage earlier healthcare utilization. Additional recommendations included improving working conditions for medical staff, expanding professional training opportunities, and establishing specialized ophthalmology units for conditions such as glaucoma and pediatric eye diseases.

Overall, participants emphasized that improving ophthalmological care requires coordinated efforts to strengthen workforce capacity, enhance infrastructure and technology, and promote preventive health education among the population.

These recommendations highlight that improvements require system-level interventions rather than isolated changes, emphasizing the need for coordinated health system strengthening.

## Discussion

We found that public awareness of eye diseases was generally perceived as moderate, and respondents noted that prevention activities were mainly directed toward older adults and children, with weaker reach among working-age adults. Participants also described limited population-level educational programming, challenges in coordination of preventive initiatives, and delayed care-seeking behavior. These findings are consistent with prior research showing variation in awareness of common eye diseases and highlighting the role of sociodemographic factors in shaping knowledge and service use (11–13). Importantly, these patterns should be interpreted within the context of Kazakhstan’s healthcare system. Studies conducted in Kazakhstan have suggested that national screening programs may have limited coverage and may rely on approaches that could reduce their effectiveness in early detection. These findings support our observation that preventive services are not fully reaching all population groups, particularly working-age adults (14). Interventions that provide tailored information and structured follow-up have been shown to improve patient knowledge and attitudes, including in glaucoma-related care (15). In underserved settings, integrating eye disease detection programs into primary care may improve early case identification and reduce preventable vision loss (16).

Our study highlighted a shortage of ophthalmologists, leading patients to seek care in urban medical centers or hospital emergency departments due to insufficient specialists in PHC. Similarly, the Health Resources and Services Administration projects a significant shortage of ophthalmologists by 2035, with a 12% decrease in supply and a 24% increase in demand, resulting in a 30% workforce inadequacy, particularly in nonmetro areas (17). The uneven distribution of ophthalmologists across different areas was identified not only in our study but also by Hong et al., who highlighted significant inequality in the distribution of ophthalmologists in Latin American countries, with a concentration in more developed regions (18). Although the degree of influence of this problem on various ophthalmological specialties remains insufficiently studied, the existing literature confirms the shortage of doctors in such areas as oculofacial plastic surgery, cataract surgery and treatment of pediatric glaucoma (6, 19). Consequently, the shortage of specialists leads to longer wait times for ophthalmological care and delayed treatment initiation, a barrier to service access identified in both our study and international research (20–21). Similar workforce and access-related challenges have been reported in Kazakhstan in other areas of healthcare, where limited specialist availability, delays in diagnosis, and uneven distribution of services contribute to barriers in care access (22). This suggests that, from the perspective of participating ophthalmologists, the challenges identified in ophthalmological care may reflect broader systemic constraints rather than isolated specialty-specific issues.

While this study focuses on ophthalmologists’ perspectives, the findings also point to broader workforce challenges that may not be fully addressed through expanding ophthalmologist capacity alone. In many health systems, optometrists and other primary eye care providers play a key role in delivering frontline services, including vision screening, refractive care, and early detection of eye diseases. The development and integration of an optometry workforce may therefore represent a more scalable and cost-effective approach to strengthening primary eye care, particularly in settings with limited specialist availability. Expanding multidisciplinary models of care, where responsibilities are distributed across different cadres of eye care professionals, could help reduce the burden on ophthalmologists and improve access to services at the primary healthcare level (23). However, the extent to which such models can be implemented in Kazakhstan requires further investigation, including consideration of regulatory, educational, and health system factors.

The findings of this study also highlight structural imbalances between primary healthcare and hospital-level ophthalmic services in the region. While hospital-based ophthalmic care was generally perceived by respondents as well staffed and professionally competent, primary healthcare facilities often face shortages of both specialists and diagnostic equipment. Participants described a structural imbalance between primary healthcare and hospital-level ophthalmic services. This imbalance may contribute, from their perspective, to the concentration of services in urban centers and increased reliance on hospital-based care for conditions that could potentially be detected earlier at the primary care level. As a result, the role of PHC as a gatekeeper for early detection and monitoring may be perceived as weakened, potentially contributing to delays in diagnosis and treatment. Such patterns are characteristic of post-Soviet healthcare systems, where hospital-centered models and weaker primary care integration continue to influence referral pathways and service utilization.

Similar disparities between urban and rural ophthalmic services have been reported in several middle-income countries, where specialized medical services tend to be concentrated in major urban centers. These findings suggest that the challenges described by participants may reflect broader structural issues affecting ophthalmic care delivery in geographically large health systems with uneven resource distribution. Understanding how workforce allocation, infrastructure availability, and health system organization influence access to ophthalmic services is therefore important not only for Kazakhstan but also for other countries seeking to strengthen eye care within PHC systems.

The lack of equipment and new healthcare technologies affects care quality. Jacques le Roux et al. found that a quality improvement project in the Accident and Emergency department enhanced the availability of ophthalmological equipment, saving clinician hours, improving patient care and flow, and showing that similar interventions across the NHS could save over 700,000 clinician hours annually (24). In our study, highlighted limitations in equipment at the primary healthcare level, suggesting that targeted assessment and potential improvements in equipment availability may be beneficial for improving timely care and optimizing resource use. Although Kazakhstan has established a process for evaluating health technologies, physician awareness remains limited, and managers could play a crucial role in promoting the adoption of these innovations (25, 26). Introducing new technologies at the regional level would also drive innovation and address stagnation in eye care services.

Overall, the findings highlight, from the perspective of ophthalmologists, the perceived importance of strengthening PHC capacity, improving workforce distribution, and enhancing preventive ophthalmic services as potential components of a more equitable and effective eye care system.

### Limitation and future research

This study included 22 ophthalmologists, providing meaningful coverage for qualitative inquiry within the East Kazakhstan region. Some participants had experience working in both hospital and primary healthcare settings, allowing for a broader perspective on ophthalmic care across different levels of the healthcare system.

However, several limitations should be acknowledged. First, participants expressed reluctance to have interviews audio-recorded, which may have limited the capture of nuanced expressions or fully verbatim quotations. To address this, detailed written documentation was completed during each interview, and responses were immediately reviewed and confirmed with participants to ensure accuracy.

Second, the study was conducted in a single region of Kazakhstan, which may limit the transferability of findings to other regions with different healthcare structures and population characteristics. Third, detailed demographic information (e.g., age, sex, and workplace location) was not systematically collected, limiting the ability to further contextualize participants’ perspectives. Finally, the inclusion of only one professional group (ophthalmologists) means that the findings reflect provider perspectives rather than a comprehensive health system assessment; perspectives from patients and other stakeholders were not explored. The lack of detailed demographic characteristics (e.g., age and sex) limits the ability to assess how perspectives may vary across participant subgroups.

Future research should expand to include a wider range of healthcare professionals and regions across Kazakhstan to improve generalizability. Incorporating patient perspectives would provide a more comprehensive understanding of barriers to accessing ophthalmological care. In addition, longitudinal studies are needed to evaluate the long-term impact of interventions such as early screening programs, improved equipment availability, and enhanced specialist training. Further research should also explore the integration of digital health solutions and emerging technologies in ophthalmic care. Collaboration with policymakers will be essential to translate these findings into practical strategies aimed at improving the accessibility, quality, and equity of ophthalmological services.

## Conclusion

Critical gaps in ophthalmological care in East Kazakhstan, particularly in primary healthcare settings, include insufficient public awareness, a shortage of specialists, and limited access to necessary equipment and technologies. The findings underscore the importance of strengthening public education on eye health, expanding specialist training, and improving the distribution of ophthalmologists across the region. These findings suggest, from the perspective of participating ophthalmologists, that targeted interventions, such as strengthening public education, expanding specialist training, and improving workforce distribution, may help improve access to ophthalmological care in the region. Collaboration with policymakers to improve the availability and integration of new technologies in the healthcare system will be essential for ensuring equitable access to quality ophthalmic care.

## Data Availability

The raw data supporting the conclusions of this article will be made available by the authors, without undue reservation.
